# Supplementary material for: Are carbon emissions trading and green financial instruments synergistic? -Comprehensive quantitative research based on content analysis
Source: PLoS One. 2024 Mar 7;19(3):e0298601. doi: 10.1371/journal.pone.0298601 (PMC10919601; doi:10.1371/journal.pone.0298601)
Supplement: S1 Data — (ZIP) [file pone.0298601.s001.zip › Data/地级市Z_F工作报告.zip!/─┌├╔╣┼/╬┌└╝▓∞▓╝╩╨/└┤╫╘╣1⁄2╓┌║┼í╛┬φ┐╦╩2╛▌═°í┐_2018.docx]

各位代表：

本届政府于2013年1月履职，任期即将结束。现在，我代表市人民政府向大会报告工作，请予审议，并请市政协委员提出意见。

一、过去五年回顾和2017年主要工作

过去五年，是我市发展史上极不平凡的五年。五年来，在以习近平同志为核心的党中央和自治区党委政府、市委的坚强领导下，全市各族人民守望相助，团结奋斗，在全面建成小康社会的伟大征程中，凝心聚力，攻坚克难，圆满完成“十二五”规划目标，顺利实施“十三五”发展规划，干成了一批事关长远发展的大事要事，实现了一系列具有标志性意义的重大突破，经济社会发展取得了令人鼓舞的重大成就。我们成功创建国家园林城市、国家卫生城市，我们无比自豪地步入“航空时代、高铁时代、大数据时代和大物流、大旅游、大开放时代”，开启了乌兰察布现代化建设的新征程。

这是综合实力大幅提升的五年。五年来，我们积极应对经济下行压力，全力以赴抓发展、稳增长，实现了新常态下的新发展。预计全市地区生产总值达到835亿元，年均增长7.3%；一般公共预算收入达到43亿元，年均增长4.4%；社会消费品零售总额达到344亿元，年均增长9.9%；固定资产投资达到542亿元，年均增长6.5%。五年累计实施亿元以上重大项目1001个，完成投资2082亿元，是上一个五年的1.3倍。转方式调结构取得新进展，农牧业现代化水平不断提高，大数据、新能源、新材料等战略性新兴产业快速成长，现代服务业规模和效益同步增长，三次产业结构由15.6:53.8:30.6优化为12.5:49:38.5，经济发展不断向着更高质量、更有效益、更可持续方向迈进。

这是城乡面貌深刻变化的五年。五年来，我们秉持“以人为本、尊重自然”的理念，统筹兼顾抓基础、搞建设，全民动员推进“五城联创”，共建美好家园。城市面貌日新月异，公用绿地面积、人均道路面积、建成区绿化率均跨入自治区前列，创建成8个自治区园林县城、3个自治区卫生县城、3个全国和谐社区。城镇化率由43.6%提高到47.9%。美丽乡村展现新姿，1321个嘎查村的基础设施条件得到改善，27个村镇分别荣获“全国文明村镇”“国家美丽村镇”等称号。立体交通体系更加完善，高等级公路里程居全区第二，嘎查村实现沥青水泥路全覆盖，乌兰察布机场建成通航，乌兰察布至呼和浩特高铁开通运营。大容量专用光缆直通北京，电网、水利等设施得到加强。生态建设和环境保护力度空前，累计投入160亿元，完成林业生态建设419万亩，森林覆盖率达到23.38%，超过自治区平均水平。污染物排放总量、能源资源消耗强度明显下降，一批突出环境问题得到有效整治。良好的生态、清新的空气成为乌兰察布一张靓丽名片。

这是改革开放全面深化的五年。五年来，我们以勇于创新、敢于担当的精神，群策群力抓改革、争试点，抢抓机遇、借势借力，推出一批有力度、有特色、有影响的改革举措。建成全区首个市场诚信平台和首家权力监督管理办公室，企业和群众办事时间压缩一半以上。四子王旗被确定为全区唯一的县级国家主体功能区试点，4个旗县列入国家重点生态功能区。中心城区“多规合一”全面启动，城市管理综合执法体制改革顺利推进。农村养老、脱贫车间、健康扶贫、创业创新、外出务工服务体系等一批独具特色的改革举措成为全区亮点。教育三项改革成效显著，管办评分离改革成为全国试点。全方位开放格局加快形成，乌大张合作区建设上升为国家战略，承接北京产业转移取得新成果。中欧班列铁路枢纽节点城市成功获批，在“一带一路”建设中的地位更加凸显。大开放、大合作成为乌兰察布发展的主旋律、新动力。

这是人民生活显著改善的五年。五年来，我们始终坚持问题导向、民生导向，持之以恒重投入、促和谐，财政用于民生的支出占公共预算支出的83%以上，城乡居民人均可支配收入年均增长8.7%和10%，超过经济增速。贫困人口减少到3.8万户、8.4万人，贫困发生率下降到4.2%。累计新增城镇就业8.5万人，农村牧区劳动力转移就业稳定在70万人。覆盖城乡的社会保障体系基本建立，保障标准逐年提高。城乡医疗条件显著改善，市中心医院升级为三甲医院。文化事业繁荣发展，一批重大文化场馆投入使用。竞技体育、全民健身协调推进。平安乌兰察布建设深入开展，安全生产形势总体稳定，食品药品安全、质量安全监管力度加大。国防建设开创新局面，“全国双拥模范城”实现三连冠。人防、地震、气象、档案、史志、外事、侨务等工作得到加强，红十字、妇女儿童、老龄和残疾人事业取得新进展。各民族共同团结奋斗、共同繁荣发展的局面进一步巩固，人民群众的获得感、幸福感、安全感显著增强。

各位代表！2017年是实施“十三五”规划的重要一年。我们深入学习宣传贯彻党的十九大精神，始终保持发展信心不变、改革力度不减、攻坚步伐不停，跑出了转型升级、创新发展的加速度。我们热烈庆祝自治区成立70周年，凝聚起“建设亮丽内蒙古，共圆伟大中国梦”的强大力量。我们成功举办首届乌兰察布发展大会、“一带一路”物流论坛，合作共赢、共谋发展的朋友圈越来越大。

我们以高度的政治自觉，推进中央和自治区巡视、环保督察反馈意见的整改落实，积极防范财政金融风险，发展步伐更加坚实。

我们坚持实事求是、注重发展质量的原则，按照中央、自治区统一要求，坚决纠正多年来积累的部分经济数据虚增问题，下决心做实基数、挤掉水分。预计2017年全市地区生产总值增长5%，固定资产投资下降18%，一般公共预算收入同口径下降19.7%。做实基数后，更有利于我们夯实基础、甩掉包袱、轻装上阵。从经济运行整体情况来看，2017年工业用电量、货运量分别增长26%和12%，均保持两位数增长，公网用电量全区第一，规模以上工业产品销售率达到95%以上，就业比较充分，城乡居民人均可支配收入分别增长8%和8.5%，呈现出经济稳中向好、人民生活水平持续提高的良好势头，积极因素和新动能正在加快积聚。

一年来，我们主要做了以下工作：

一是突出项目带动，加快推动产业转型。全面打响重大项目建设攻坚战，实施亿元以上项目286个，完成投资398亿元。出台IPO、旅游、金融、大数据等一揽子优惠政策，在北京、上海、深圳等地举行招商推介会，引进项目181个，到位资金230亿元，一批事关未来发展的大项目、好项目相继落地，投资兴业的优势更加凸显。抢占发展先机，大数据产业异军突起。苹果、阿里等数据中心项目成功落户，华为二期开工建设，华唐、博岳、鸿联九五、软通动力等企业加快集聚，打造呼叫中心坐席5000席，可带动直接就业1万人，奠定了“南贵北乌”大数据中心地位。发挥区位交通优势，大物流迅速发展。天津港物流基地、曹妃甸内陆港挂牌成立，庙梁物流园区“三线一站”开通运营，博源数字化配煤项目建成投用，国家煤检中心获准授权。集宁现代物流园区荣获“全国示范物流园区”称号。七苏木中欧班列物流基地启动运行。中欧班列开行70列，返程15列，带动木材、皮件等产业迅速发展。北方家居产业园19家企业开工建设，中国新雅宝路商城订单总额达到2.5亿元。山东顺和等一批国内先进物流企业在我市签约投资。抢抓冬奥会和高铁开通机遇，大旅游方兴未艾。岱海滑雪场、红召九龙湾生态旅游区建成运营，格根塔拉完成改造提升工程，集宁战役纪念地入选全国红色经典景区。成功举办中俄蒙美食文化节、草原文化音乐节、草原巴哈大赛、国际冰雪艺术节等重大活动。全市4A级景区增加到10个，游客数量和旅游收入分别增长16%和25%，全域旅游让乌兰察布四季都是好时节，“京郊草原、避暑之都”品牌更加响亮。发挥资源能源优势，新能源新材料形成规模。风电、光伏并网装机达到595万千瓦，占全区的五分之一。中聚锂电池等项目开工建设，新能源电池产能不断扩大。冶金、化工等资源型产业实现延伸升级，建成全国单体最大、技术最先进的PVA生产基地。吉林铁合金整体迁入，我市成为全国最大的铁合金生产基地。发挥绿色生态优势，现代农牧业发展步伐加快。薯菜乳肉等优势主导产业不断壮大，“乌兰察布燕麦”等18种产品获得国家地理标志认证，我市成为全国优质种薯黄金产区、中国燕麦主产区、全国三大冷凉蔬菜基地之一、内蒙古牛羊肉重要生产基地。农畜产品加工转化率达到59%。“中国燕麦之都”正在申报，“乌兰察布马铃薯”荣获中国百强农产品区域公用品牌，鸿茅药酒入选央视国家品牌计划。

二是突出城乡融合，持续改善环境品质。以“五城联创”和“美丽乡村”建设为抓手，人居环境明显改善。投资94亿元，实施南沙河综合治理等市政建设项目22个。开工棚户区改造2.74万套，超额完成自治区任务。积极回应群众呼声和关切，政府筹资27亿元，启动“半拉子”工程，下决心解决中心城区回迁安置历史遗留问题，一批逾期多年的回迁户得到妥善安置。农危改年度任务全部完成，改善了3万户农牧民居住条件。土牧尔台镇、岱海镇入选全国特色小镇，察右前旗南村成为全国农村人居环境保障示范村。以环保督察整改为契机，强力推进生态环境保护。中央、自治区环保督察反馈意见整改工作有序推进。岱海、黄旗海水生态保护规划获得自治区政府批复，环岱海16万亩耕地完成水改旱，岱海电厂水冷改空冷技改工程开工建设。黑河治理工程取得阶段性成效。自然保护区工矿企业清理整治专项行动全面实施。城镇污水处理厂全部提质升级，污水集中处理率达到95%，中心城区全面取缔10蒸吨以下小锅炉。完成林业生态建设64万亩，白泉山山体修复暨公园建设项目荣获中国人居环境范例奖。以构建大交通格局为重点，基础设施走向互联互通。实施重大交通项目42项，完成投资65.5亿元。集宁至科布尔等9条公路全线通车，6条旅游公路交付使用。乌兰察布高铁车站建成投用，到呼和浩特的时间缩短至39分钟。乌兰察布机场开通航线10条，通达20个国内主要城市，年旅客吞吐量突破30万人次。

三是突出改革创新，全面释放发展活力。供给侧结构性改革深入推进。淘汰钢铁过剩产能170万吨。商品房去库存2.5万套，去化周期由24个月压缩至14个月，库存量处于合理区间。取消免征停征49项行政事业性收费，实施电力多边交易、“营改增”等政策，企业用电成本和税费负担下降27.7亿元。全面规范政府债务管理，防范财政金融风险，停建缓建了一批可能新增债务的项目。重点领域改革取得突破。习近平总书记要求内蒙古先行先试的三项改革深入推进。81%的龙头企业与农牧民建立利益联结机制，40万农牧户进入产业化经营链条。生态文明制度改革取得积极成效，《水资源保护条例》颁布实施，“河长制”全面建立，草原确权基本完成。同俄蒙合作机制不断深化，中欧班列过货量达到6.2万吨，实现去程汽车、日用品与返程木材等商品的双向流动。全面深化“放管服”改革，市本级“权责清单”全部公开，审批环节减少27%。实行区域综合评价评估和联合审图、联合验收，为重大项目落地开通“绿色通道”。深入实施“多证合一”登记制度改革，新增各类市场主体2.3万户，平均每个工作日注册超过90户。创新投融资体制机制，岱海滑雪场等一批PPP项目启动实施。开放合作水平明显提升。成功举办第四届乌大张合作区联席会议，各领域合作不断深化。实施京蒙对口帮扶项目27项，43家IPO排队上市企业在我市注册。外贸进出口备案企业新增64家、达到375家。外贸进出口总额2.5亿美元，增速全区第一。

四是突出共建共享，切实增进民生福祉。脱贫攻坚取得阶段性成果。出台加大脱贫攻坚力度的十项措施，4.7万贫困人口实现脱贫。在全区率先推行“慢病送药、大病兜底、一站式服务”的健康扶贫模式，贫困人口大病住院和慢病送药报销比例超过90%。累计撤并30户以下小村1162个，开工建设移民安置住房3.3万套。推广政融保、菜单式扶贫、入股分红、公益乌兰察布和脱贫车间等产业扶贫模式，带动贫困户稳定增收。“双创”新引擎作用凸显。与新华网合力打造的中国创业创新博览会永久落户我市。建成国家级众创空间3个，集宁区入选国家级返乡创业试点地区。科技创新能力明显增强，建成“一园两院五站十四中心”，蔬菜品种自主知识产权实现零的突破。组建北方大数据学院，实现保障就业与扶持产业互促互动。社会保障机制更趋完善。社会养老保险实现制度全覆盖，职工和城乡居民待遇每月分别提高127元和20元。城镇职工和城乡居民医疗保险政策范围内报销比例提高10个百分点，分别达到90%和80%。城乡低保、特困人员供养、困境儿童救助标准进一步提高。社会事业持续较快发展。学前教育三年毛入园率达到95%以上。11个旗县市区全部通过国家义务教育均衡发展验收，巩固率达到98%以上。文化事业繁荣发展，《乌兰夫》系列剧—《热土—影像中的珍贵记忆》在京首映，《忠勇察哈尔》《满都海斯琴》等多部精品深受赞誉。察右后旗乌兰牧骑荣获自治区金奖。健康乌兰察布扎实推进，城市公立医院改革试点工作取得实效，国家食品安全示范城市创建工作走在全国前列。游泳馆、网球馆等场馆建设加快推进，全区第十四届运动会筹备工作有序进行。新建标准足球馆2个、笼式足球场25个、夜间篮球场14个，全部免费向群众开放。社会治理水平有效提升。严格落实安全生产责任制，实现事故起数、死亡人数“双下降”。立体化治安防控体系不断完善，信访维稳工作得到加强，圆满完成重大活动安保任务。

五是突出自身建设，提升施政服务水平。打铁必须自身硬。我们坚持把全面从严治党要求贯穿于政府工作各个方面，持续开展“两学一做”学习教育，认真落实巡视反馈问题整改意见，制定实施“三重一大”集体决策等制度，政府系统廉洁建设全面加强。加大行政监察和审计监督力度，严肃查处各类违纪违法案件。政府预算、部门预算、三公经费全部向社会公开。强化专项督查，狠抓重大政策落实。认真执行人大及其常委会的决议决定，自觉接受人大、政协和社会监督。人大代表议案、建议和政协委员提案办复率达到100%，3件地方性法规议案通过市人大常委会审议。

各位代表！回首五年，我们付出了艰辛，实现了跨越，每个乌兰察布人都为之振奋和自豪。这是以习近平同志为核心的党中央亲切关怀的结果，是自治区党委、政府和市委坚强领导的结果，是市人大、市政协大力支持的结果，是全市各族人民团结奋斗、苦干实干的结果。在此，我代表市人民政府，向在各条战线上辛勤工作的各族干部群众，向人大代表、政协委员，向各民主党派、工商联、无党派人士和各人民团体，向解放军、武警官兵、政法干警，向所有关心、支持和参与乌兰察布建设发展的同志们、朋友们，致以崇高的敬意和衷心的感谢!五年的砥砺奋进，不仅为未来发展奠定了更加坚实的基础，也积累了十分宝贵的经验。最根本的一条，就是要牢固树立“四个意识”，坚决同以习近平同志为核心的党中央保持高度一致，不折不扣地贯彻执行中央大政方针和决策部署。实践充分证明，成绩是干出来的，必须始终坚持发展第一要务，必须始终把人民放在心中最高位置，必须解放思想、扎实苦干，才能干出乌兰察布发展的新境界，干出人民满意的新业绩。

成绩值得肯定，问题不容回避。我们最突出的问题就是发展不平衡、不充分的问题，这已经成为满足人民日益增长的美好生活需要的主要制约因素。不充分主要表现在：经济总量小，产业结构不合理，一二三产融合不充分。现代农牧业经营体系不健全，龙头企业不多，品牌创建意识不强。传统产业生产方式粗放，生产性物流、仓储、电商等配套体系不完善，服务业发展水平不高。招商引资精准性不够，经济增长还缺乏大项目支撑和强有力的人才支撑。政府债务化解压力大，收支矛盾突出。实体经济融资难问题依然存在。不平衡主要表现在：农村牧区发展欠账多，脱贫攻坚任务艰巨。公共服务水平低，人民群众关心的就业、上学、看病、物业管理等方面还存在不少问题，城市管理水平有待提高。生态环境保护任重道远，资源环境约束日益凸显，全民节水意识和机制没有真正形成，安全生产还有不少隐患。同时，政府工作还有很多不足，一些干部为企业主动服务意识不强、落实不力，为群众解决问题效率不高、作风不实，“办事难”现象还在一定程度上存在。特别是在打造旅游城市过程中，人性化服务管理水平不高，热情好客、开放包容的城市形象还没有真正树立起来。对此，我们要高度重视，始终坚持问题导向，坚决克服弊端，加快补齐短板，尽心竭力做好政府工作，决不辜负人民群众的信任和期望。

二、今后五年总体思路和目标任务

今后五年，是实现“两个一百年”奋斗目标的历史交汇期。我们将迎来改革开放40周年、新中国成立70周年、全面建成小康社会和中国共产党成立100周年等重要时间节点。做好今后五年工作，责任重大，意义深远。

党的十九大确立了习近平新时代中国特色社会主义思想的指导地位，描绘了全面建设社会主义现代化强国的宏伟蓝图，这是我们开启新征程、续写新篇章的行动纲领；新一轮科技革命、产业变革蓬勃兴起，国家“一带一路”、京津冀协同发展和乡村振兴、脱贫攻坚等一系列重大政策深入实施，这是我们千载难逢的宝贵机遇；尤为重要的是，经过近几年的艰苦奋斗，我市区位交通、能源资源、绿色生态、产业基础、城市配套等方面的比较优势更加凸显，新的经济增长点不断形成，发展后劲明显增强，发展前景十分广阔，这是我们砥砺前行的重要基础。我们要准确把握历史新方位，抢抓时代新机遇，发挥优势、乘势而上，一件事情接着一件事情办，一年接着一年干，锲而不舍向前走，努力在新一轮区域竞争中实现后发赶超、跨越发展。

按照中共乌兰察布市第四次党代会和市委四届六次全会的安排部署，新一届政府工作的总体思路是：全面贯彻落实党的十九大精神，以习近平新时代中国特色社会主义思想为指导，统筹推进“五位一体”总体布局和协调推进“四个全面”战略布局，认真贯彻新发展理念，坚持稳中求进工作总基调，按照高质量发展要求，以供给侧结构性改革为主线，统筹推进稳增长、促改革、调结构、惠民生、防风险各项工作，坚决打好防范化解重大风险、精准脱贫、污染防治“三个攻坚战”，奋力夺取决胜全面建成小康社会伟大胜利。

各位代表！实现梦想,时不我待。从现在到2020年，是全面建成小康社会决胜期，仅有短短的3年时间，任务繁重艰巨。全市还有8.4万人没有脱贫，全面建成小康社会40项指标还有26项没有达标。贫困不除、愧对历史，群众不富、寝食难安，小康不达、誓不罢休。我们必须付出百倍的努力，紧盯目标、精准发力，以最后冲刺的劲头和勇于担当的精神，突出抓重点、补短板、强弱项，确保到2020年与全区、全国同步全面建成小康社会，到2022年实现经济社会更高水平、更高质量发展。

——坚持新发展理念，在推动高质量发展中实现新突破。推动高质量发展是当前和今后一个时期确定发展思路、制定经济政策的根本要求。要以深化供给侧结构性改革为主线，坚持质量第一、效率优先，加快构建现代化经济体系。运用高新技术和先进适用技术,改造提升化工、冶金、建材、农畜产品加工等产业，让传统产业焕发新活力、增强竞争力。加快培育大数据、高端装备制造、新能源、新材料等优势产业集群，到2019年建成1200万千瓦新能源基地，到2020年数据中心服务器达到100万台，主营收入超过百亿元。通过五年的不懈努力，到2022年力争建成国家级大数据产业基地和“草原云谷”，新材料、高端装备制造产业形成规模，战略性新兴产业增加值占GDP比重达到10%以上。下大力气抓好物流、旅游、养老、会展、金融等现代服务业，建成高铁商圈、临空产业园、综合保税区，打造华北地区重要的物流集散基地，形成连通京津冀港口和俄蒙欧腹地的国际物流通道，构建全面开放新格局。打造国内外知名的全域旅游目的地，旅游人数和收入年均增长20%左右。服务业在国民经济中的比重达到45%以上，经济结构更加合理。深入推进农牧业供给侧结构性改革，补齐加工、销售等短板。积极发展旱作农业、节水农业、设施农业，种植业要培育壮大马铃薯、冷凉蔬菜、燕麦杂粮杂豆等产业，养殖业要稳羊增牛扩猪禽，着力构建粮经饲统筹、农林牧结合、种养加一体、一二三产融合的发展机制。大力实施乡村振兴和区域协调发展战略，推动城乡要素自由流动、平等交换、合理配置，加快形成工农互促、城乡互补、全面融合、共同繁荣的新型工农城乡关系，县域经济实力明显增强，美丽乡村建设让农牧民的生活更有奔头。城镇化率达到55%以上。全面加强铁路、公路、航空、市政、水利、电力、信息等基础设施建设，着力解决水、电、气短缺等问题，提高城乡综合承载能力，为经济社会长远发展奠定坚实基础。

——坚持突出工作重点，在打赢三大攻坚战中取得新成果。打赢三大攻坚战是中央的重大决策部署，也是我市全面建成小康社会必须跨越的关口。坚决打赢防范化解重大风险攻坚战。将政府债务化解纳入实绩考核体系，积极化解存量，严格控制增量，强化预算约束，严格规范政府举债融资行为，到2021年政府债务率控制在合理区间，坚决杜绝寅吃卯粮、透支未来。加大处置金融风险工作力度，提高金融服务实体经济能力，力争五年内不良贷款率低于全区平均水平，守住不发生系统性区域性风险的底线。坚决打赢脱贫攻坚战。我市是自治区脱贫攻坚主战场，必须把脱贫攻坚作为头等大事和第一民生工程，以脱贫攻坚统揽经济社会发展全局，所有工作都要向脱贫攻坚倾斜，举全市之力打赢这场硬仗。确保到2020年现行标准下农村牧区贫困人口实现脱贫，贫困旗县全部摘帽。坚决打赢污染防治攻坚战。牢固树立“绿水青山就是金山银山”的理念，坚守生态底线，不碰环保红线，像爱护生命一样爱护生态，守护好我们最大的优势、最宝贵的财富。坚持以水定城、以水定人、以水定产，强化节约用水。统筹山水林田湖草系统治理，全力解决好大气、水、土壤等环境突出问题，决不以牺牲环境、浪费资源为代价换取一时的经济增长。森林覆盖率达到25.1%以上。单位地区生产总值能耗、主要污染物排放控制在国家下达的指标以内，推动形成人与自然和谐发展的现代化建设新格局，让乌兰察布山水“颜值”更高、大地“气质”更佳。

——坚持以人民为中心，在保障和改善民生中谱写新篇章。发展的目的是让全市人民过上美好生活。新一届政府将以造福人民为最大政绩，始终同人民想在一起、干在一起，多谋民生之利、多解民生之忧，在增进民生福祉、促进人的全面发展上作出更加有效的制度安排，使发展更有温度、幸福更具质感。坚持民生支出水平随经济增长同步提高，持续增加公共服务供给，着力在发展中补齐民生短板、促进社会公平正义。积极推进大众创业、万众创新，城乡居民收入增速快于经济增长。全面完成城市棚户区和农村牧区危房改造，城乡人居环境得到明显改善。统筹各类教育协调发展，教育教学质量显著提升。持续深化医药卫生体制改革，打造1-2个自治区领先学科，每个家庭拥有一名签约医生，每个居民拥有一份电子化健康档案。免费开放的文化体育公共服务设施逐年增加。覆盖城乡的社会保障制度更加完善。民族团结、社会稳定、边疆安宁的政治局面得到全面巩固和发展，乌兰察布成为各族人民安居乐业的幸福家园。

各位代表！发展全凭实干，奋斗成就梦想。只要全市各族人民坚定信心、苦干实干、顽强拼搏，就一定能够用我们的智慧和汗水，创造出不负历史、无愧时代的辉煌业绩，赢得全面建成小康社会的最后胜利！

三、2018年重点工作

五年看头年，开局最关键。2018年，是新一届政府的开局之年，也是贯彻党的十九大精神的开局之年，是决胜全面建成小康社会、实施“十三五”规划承上启下的关键一年。我们要着眼五年、立足当年，突出重点、把握关键，努力开创乌兰察布改革发展新局面。

今年经济社会发展主要预期目标是：全市地区生产总值增长7%左右，固定资产投资增长10%左右，一般公共预算收入增长5%左右，社会消费品零售总额增长8.5%左右，城乡居民人均可支配收入分别增长8%和8.5%左右，城镇新增就业1.65万人左右，城镇调查失业率和城镇登记失业率分别控制在5%左右和4%以内，单位地区生产总值能耗完成国家下达的目标，供给侧结构性改革取得实质性进展，政府债务化解完成年度任务，财政金融风险得到有效防控。确定上述发展目标，体现了稳中求进的总基调，体现了更加注重提高发展质量和效益的导向，注重与全面建成小康社会目标的有机衔接。按照中央、自治区要求，首次把城镇调查失业率纳入预期目标，主要是考虑这一指标涵盖农民工等城镇非户籍人口，能够更全面准确地反映就业情况。

今年要重点抓好以下工作：

（一）大力实施乡村振兴战略，集中力量打好脱贫攻坚战

“三农”向好，全局主动。要坚持农业农村优先发展，把脱贫攻坚与乡村振兴战略紧密结合起来，按照“产业兴旺、生态宜居、乡风文明、治理有效、生活富裕”的总要求，加快推进农业农村现代化。

以“绣花”功夫抓好精准脱贫。坚持把脱贫质量放在首位，严格落实“六个精准”要求，统筹处理好贫困户与非贫困户、输血与造血、扶贫与扶志扶智、继续攻坚与防止返贫的关系，举全市之力攻克深度贫困堡垒。年内实现4.5万贫困人口稳定脱贫。围绕“两不愁”合力攻坚。突出产业扶贫。进一步完善订单带动、入股分红等农企利益联结机制，继续推广脱贫车间模式，选准主攻产业和主攻方向，因地制宜发展特色农牧业、旅游、电商等产业，实现户户有增收项目、人人有脱贫门路。实施好“互联网+乡村”信息平台和公益乌兰察布项目，大力开拓农畜产品市场，建立稳定销售渠道，增加贫困户收入。加快建设光伏扶贫电站，不断壮大集体经济。抓住保监会对口帮扶机遇，引入保险资金，加大金融扶贫力度。强化兜底保障，推动农村牧区低保与扶贫开发双向衔接，做到“应扶尽扶、应保尽保”。围绕“三保障”合力攻坚。做好搬迁对象认定、工程建设和资金使用管理等工作，基本完成小村整合和易地搬迁任务，全面完成农村牧区危房改造。推进教育扶贫，健全完善贫困学生资助体系，落实好从学前教育到高等教育的助学政策。抓好健康扶贫，实现贫困人口送医送药签约服务和免费健康体检全覆盖。根据我市常见慢性病种，修改完善慢性病用药指导目录，增加报销品种，扩大报销范围，减轻贫困户看病支出。贫困人口大病住院和慢病送药报销比例达到95%。围绕“长效机制”合力攻坚。强化市县党委政府主体责任，发挥好包村干部、驻村工作队、第一书记作用，帮助贫困群众把种什么、养什么、从哪里增收、怎么脱贫想明白做到位。深化京蒙对口帮扶，支持好中央和区直单位定点扶贫，积极鼓励民营企业参与精准扶贫。加大涉农涉牧和扶贫资金统筹整合力度。加强扶贫领域作风问题专项治理，力戒形式主义，坚决防止弄虚作假，杜绝数字脱贫，切实做到真脱贫、脱真贫。

构建现代农牧业三大体系。以市场需求为导向，坚持走特色路、打绿色牌，持续深化农牧业供给侧结构性改革，为消费者提供更丰富、更适销对路的农畜产品，提供亲近自然、回归乡村的消费体验和服务。“调优”农牧业产业体系。建设祖国正北方冷凉经济带，打造面向首都的绿色农畜产品生产供应基地。抓住马铃薯主粮化机遇，以做强种薯产业为方向，以培育引进深加工企业为重点，推动马铃薯全产业链发展，实现由量的扩张向质的提升转变，真正让“中国薯都”的品牌叫响全国。新增优质专用薯30万亩、达到180万亩，形成150万吨种薯生产能力。大力发展设施农业，冷凉蔬菜种植面积发展到100万亩，其中设施蔬菜23万亩。壮大杂粮杂豆产业，开发燕麦系列产品，打造“中国燕麦之都”。鼓励发展旱作特色农业，抓好藜麦、中草药等经济作物种植。因地制宜推进经济林建设，新增面积不低于10万亩。适度规模发展生猪产业，发挥雏鹰、朋诚、大北农等企业带动作用，打造6个百万口生猪大县。加强肉羊标准化养殖场建设，打造杜蒙肉羊生产基地，提高养殖品质和效益，支持企业提升就地加工转化能力。推动肉鸡、肉牛和驴产业发展，多渠道带动农牧民增收致富。大力发展草产业，建设高产优质饲草基地150万亩。“调绿”农牧业生产体系。充分考虑土地、水、环境等资源约束，合理确定种养规模、品种和区域，推动农牧业绿色发展。深入开展控肥、控药、控膜、控水“四控行动”。抓好牲畜粪污无害化处理，综合利用率达到70%以上。推进农业水价综合改革，逐步建立水价形成、精准补贴和节水奖励机制。大力发展节水农业，压减高耗水作物种植，节水灌溉面积达到255万亩。“调强”农牧业经营体系。坚持以市场引导生产，紧扣营销发力，完善仓储设施和物流配送体系，扩大订单式生产。整合扶贫资金，高标准建设马铃薯等农产品仓储设施，新增仓储能力30万吨，资产量化折股到贫困户，企业承租运营，贫困户收益分成。建设一批蔬菜恒温库，健全冷链物流运输体系，扩大冷凉蔬菜市场占有率。引进培育一批产业化龙头企业，突破“原字号”、改变“初加工”，进一步延长产业链、提升价值链、拓宽增收链。加工转化能力达到62%。争取5个国贫县列入国家电子商务进农村示范县，实现电商快递综合服务网点行政村全覆盖。实施品牌发展战略，培育一批在全国立得住、叫得响、卖得好的农畜产品品牌。

深化农村牧区改革。着力整合各种土地资源，积极化解土地承包矛盾纠纷，理顺承包关系，完成土地确权登记颁证工作。稳妥推进“三权”分置制度改革，真正让农户的承包权稳得住、经营权活起来。做好村集体资产、资源、资金“三资”清理工作，提升基层组织服务群众的能力。深化供销合作社改革，创建自治区级示范社20个、国家级示范社5个。发展适度规模经营，新增土地流转面积30万亩、达到515万亩。完成国有林场改革。探索开展“两权”抵押贷款试点，加快建立农村牧区产权交易流转平台。鼓励金融机构设立乡镇金融服务站，加大对农牧民金融需求的支持。

推进美丽乡村建设。巩固提升农村牧区建设成果。大力改善水电路讯房等基础设施，建好管好护好运营好农村公路。开展农村环境综合整治，实施农村牧区垃圾处理设施建设工程，推进“厕所革命”。完成饮水安全工程建设任务。大力发展休闲农业、体验农业、田园综合体等新业态。加强历史名镇名村、传统村落的保护开发，因地制宜推进特色小镇建设，保护好绿水青山和清新清净的田园风光，让乡村留住美丽、记住乡愁。促进农村牧区文化教育、医疗卫生等事业发展，加强农村牧区基层基础工作。集中开展“关爱老人、孝顺老人”主题教育活动，健全自治、法治、德治相结合的乡村治理体系。培养造就一支懂农业、爱农村、爱农民的“三农三牧”工作队伍。

（二）大力实施创新驱动发展战略，提高实体经济质量和效益

加强创新能力建设。激发科技“第一动力”。发挥企业科技创新主体作用，鼓励企业与高等院校、科研院所合作，围绕我市主导特色产业，开展新品种、新材料、新技术科技试验示范和技术攻关。建立铁合金和石墨新材料研究院，加快高端产品和先进工艺研发，促进创新成果与市场对接，打通向现实生产力转化的通道。用好人才“第一资源”。围绕大数据、物流、旅游等优势产业，落实好我市人才发展规划、重点产业人才项目支持计划和高层次人才引进实施办法，加强高层次、高技能、紧缺急需人才队伍建设。高度重视企业家队伍建设，大力弘扬优秀企业家精神，营造企业家健康成长环境，培养更多具有战略眼光、开拓精神和社会责任感的优秀企业家。突出质量“第一标准”。实施质量强市战略，开展质量提升行动，建立完善各领域质量标准，引入质量溯源体系，发挥好市长质量奖的引领作用，支持我市企业争创国家和自治区品牌。新增农畜产品“三品一标”认证产品40个。

推动传统产业优化升级。高端化工产业。坚持多元、延伸、升级的发展方向，不断扩大PVA、PVC、氟橡胶等中高端产能。氯碱化工、氟化工产能分别达到470万吨、50万吨。冶金产业。坚持绿色化发展方向，鼓励现有矿热炉改造升级，前旗、化德工业园区完成全密闭大型化改造，提高精炼产品比例。推动吉铁、雄伟光大等项目投产运行，铁合金综合产能达到700万吨。建材产业。支持发展绿色环保建材，推广绿色建筑和装配式建筑。推进固废利用，转化率达到45%。

发展壮大战略性新兴产业。新能源产业。重点实施风电、光伏等16个新能源项目，开工建设600万千瓦风电基地及外送通道，争取“光伏领跑者”计划项目。新材料产业。以天然石墨深加工为重点，向石墨烯等高附加值产品延伸，占领行业制高点。推进天津中聚等项目建设，不断扩大锂电池负极材料和石墨电极产能，石墨化工综合产能达到45万吨，其中锂电池负极材料6万吨。新型装备制造产业。推进风机制造全产业链发展，满足我市风电基地配套要求。打造新能源电池生产基地，发展新能源汽车整车装配。加快化成箔扩产项目建设，产能达到4000万平方米，大力发展超级电容器等下游产品。推动智造谷产业园、百度无人驾驶项目建设，打造高端制造示范应用基地。

实施“大数据引领”战略。抓住国家推动大数据产业创新发展的有利机遇，加快建设“数字乌兰察布”，打造中国北方大数据中心。做强核心业态。加快苹果、阿里、优刻得等数据中心建设，推动华为二期建成投运，积极引进国家、行业、区域数据资源，提升大数据产业集聚化水平，建设国家大数据灾备中心。服务器争取超过10万台。拓展关联业态。重点发展呼叫外包、视觉工业等产业。以华唐、博岳、鸿联九五等企业为依托，继续加大关联企业引进力度，形成1万个呼叫外包席位规模，打造乌兰察布国际服务外包基地。扩张衍生业态。发挥百度双创中心、软通动力乐业空间的孵化作用，集聚一批大数据应用企业，加快数据开放共享，推动大数据在政用、商用、民用等各个领域遍地开花。

加快发展大物流产业。建设“一带一路”和中俄蒙经济走廊先导试验区，发挥中欧班列枢纽节点城市优势，加快七苏木中欧班列物流基地建设，力争开通铁路口岸、机场临时口岸，开行中欧班列120列。积极引进“平台+路港”运营模式，推动庙梁物流园区创建多式联运示范区，实现园区与港口“重去重回”。引进国内大型物流企业，建设集仓储加工、物流配送、信息服务、综合配套为一体的集散中心。发展壮大专业市场。大力发展物流加工业，加快铁合金物流园、北方家居产业园等物流加工基地建设，提升煤炭、矿石、木材等大宗商品加工能力，提高物流附加值。

全面发展大旅游产业。围绕“京郊草原·避暑之都”旅游品牌，发挥我市天蓝水碧、空气清新的独特优势，大力发展全域旅游、四季旅游。推进旅游供给品质化。完善旅游业发展规划，引进大型企业、品牌旅行社和景区托管运营商，推进岱海、格根塔拉、辉腾锡勒、火山草原等重点景区深度开发。全面启动5A级景区和国家级旅游度假区创建工作，完善旅游标识标牌、公厕、自驾露营等基础设施。加快旅游公路建设，解决景区通达最后一公里。推进旅游业态多元化。大力发展乡村旅游，重点打造86个旅游扶贫村和14个国家传统村落，建设一批精品采摘园、特色民宿和星级农家乐，让广大农牧民在山水间种出风景、种出产业、种出财富。大力发展红色旅游，利用集宁战役、绥蒙革命、红格尔图战役等红色文化资源，增设参与性、体验性红色教育设施，建设党员干部教育培训基地。大力发展冬季旅游，开发冰雪娱乐、皮草购物、温泉体验等产品，让冷资源变成热产业。大力发展文化影视旅游，促进会展经济与旅游业融合发展，加快推进会展中心项目建设，继续办好中国创业创新博览会、中俄蒙美食文化节等会展活动，打造一批重点品牌展会。推进旅游市场规范化。建立综合执法机制，加强导游队伍建设，营造舒心、放心、安心的旅游大环境。深化区域旅游合作，全面融入京津冀“一卡通”推广体系，办好首届北京旅游合作平台大会。全年接待游客1900多万人次、实现旅游收入200亿元，分别增长20%和25%。

积极发展大健康产业。培育壮大养老养生产业。加快推进百旺、福瑞、莲德医养一体化项目，建设黄河源中国国际养老产业示范基地，大力发展蒙中医药、生物科技、健康护理等新兴产业，打响面向京津的养老养生品牌。稳步推进养老服务市场化，加大政府购买居家养老服务力度。优先开展65岁以上老年人家庭医生签约服务，在全市二级以上综合医院开设老年人“绿色通道”。大力发展体育健身产业。抓住国家实施“3亿人上冰雪”政策机遇，借助京张冬奥会、全国全区冬运会，积极发展冰雪产业。加快岱海国际滑雪场娱乐项目建设，办好第二届冰雪节和“一带一路”冰雪季、“飞雪极限”城市挑战赛等活动，推进冰雪运动进校园。加快建设红海子体育产业园、足球夏令营训练基地，每个旗县建设1个标准化足球场，大力发展校园足球。打造网球、拳击、马拉松、环湖自行车等品牌赛事，建设马产业基地。当好东道主，办好全区第十四届运动会。（三）狠抓项目建设，增强经济持续发展后劲着力构建大招商格局。坚持把优化营商环境放在招商引资的首要位置，牢固树立“营商环境就是竞争力、就是生产力”的理念，深入开展“优化发展环境年”活动，着力打造更加高效便利的硬环境和软环境。进一步健全招商机制，完善招商政策，整合招商资源，形成多层次、多渠道、多元化招商网络。积极对接京津冀、长三角、珠三角等发达地区，抓住产业转移的机遇，力争引进更多的大好高新项目，年内实现“三个一百”，即开工100个、投产100个、储备100个产业项目。

强力推进项目建设。落实重大项目领导责任制，完善项目审批“绿色通道”，建立企业投资负面清单，试行承诺制无审批管理。做实项目前期工作，缩短项目落地周期。加快基础设施项目建设，做好集张高铁建设保障工作，推动集通、集二铁路电气化扩能改造，积极推进集大高铁前期工作。加快实施白永高速等一批重点公路项目，提升公路档次，强化公路管养，完善公路网络。

全力抓好要素保障。强化能源保障。优化电网结构，提高洪峰供电能力，实施一批变电站项目，推动新一轮农村牧区电网改造升级。加快陕京四线支线管道建设，推进天然气进城入户。天然气加工能力达到15亿立方米。强化用水保障。坚持开源和节流并重，构建节水、饮水、供水“三位一体”水利基础设施体系。积极争取黄河用水指标，启动岱海跨流域调水工程。加快推进中心城区应急备用水源工程，红格尔水库竣工蓄水，头道沟、吉庆峡水库开工建设。强化信息保障。集宁—北京大容量直通光缆实现商业化运营，实施二期光缆项目。完成“无线城市”项目建设。强化土地保障。优化土地资源供给，盘活用好土地存量，增量需求按项目带土地原则供地。根据投资强度和税收效益，加大土地利用考核评价力度。强化用地监管，坚决依法处置闲置低效用地。强化资金保障。推进“县域金融工程”试点工作，健全政府性融资担保体系，扩大企业融资担保覆盖面。运用好证监会扶贫政策，支持企业利用资本市场直接融资，扶持鸿茅、雪原、民丰、田牧等后备企业上市。组织实施好PPP项目，拓宽民间资本进入渠道。

提升园区建设水平。统筹发挥临空产业园、高铁商圈和各类物流园区、工业园区等重大平台带动作用，积极承接北京产业转移和非首都功能疏解。坚持错位发展、集聚发展、循环发展，制定实施园区振兴计划，进一步明确功能定位、产业定位，引导新进企业入驻园区。推行“管委会+公司”模式，理顺园区管理体制和工作机制。制定“僵尸企业”退出清单，加大“僵尸企业”处置力度。提高园区基础设施投资强度，加快完善水电热、垃圾处理、渣场等设施建设，工业园区全部建设污水处理设施。

（四）以五城联创为载体，提升城市发展水平彰显规划特色。坚持以人为本、产城互动、城乡融合，充分发挥规划的龙头和先导作用，加快构建中心城区、县级城市、特色小镇为骨架的现代城镇体系。完成中心城区“多规合一”，并在各旗县市全面推开，形成统一的规划蓝图和信息管理平台。抓好旗县市新一轮总规实施评估和特色小镇规划工作。中心城区实现控制性详规全覆盖。认真落实国家第二批城市设计试点工作，提高建筑设计水平和质量，让好山、好水、好风光融入城市，让历史文化、风土人情植入建筑。严格规划刚性约束，确保一张蓝图绘到底。

完善城市功能。统筹抓好旧城改造与新城建设、基础设施与公共服务、地下建设与地上建设。实施地下综合管廊项目，新建改建供热、供水管道80公里和75公里，集中供热、供水普及率达到92%和97%。多渠道解决棚改资金，完成3.9万套棚户区改造任务，并解决好“半拉子”工程问题。逐步打通城区断头路，提高道路互联互通水平。加强城区停车场和公交首末站建设力度，让市民出行更便捷更畅通。

提升管理品位。开展新一轮全国文明城市创建工作。深化城市管理综合执法体制改革，持续推进市容市貌综合整治。出台《物业管理条例》，完善物业服务市场化机制，提高服务质量和水平。大力推进城市“双修”，开展绿化提升、亮化改造和山体、水系、绿地修复。建设海绵城市、国家生态园林城市、国家森林城市、国家节水城市，创建一批文明县城、园林县城和卫生县城。做好国家食品安全城市创建验收工作，各旗县市区都要建成食品加工园区和封闭式农贸市场，食品小作坊入园率和流动摊贩入市率均达到80%以上。加快全民健身设施建设，打造10分钟健身圈。把乌兰察布建设成为居者心怡、来者心悦的宜居宜业宜游的现代化城市。

（五）深化改革扩大开放，激发市场主体活力

深入推进供给侧结构性改革。巩固扩大去产能成效，严禁产能过剩行业违规新上项目。坚持“房子是用来住的、不是用来炒的”，统筹房地产去库存和棚户区改造工作，加快住房制度改革和长效机制建设，引导房地产业健康发展。大力发展直接融资，降低企业负债率。严格执行收费目录清单，继续清理规范涉企收费，持续为实体经济减负。深化电力体制改革，扩大电力多边交易规模，建成丰镇氟化工园区增量配电网试点项目。加大公共服务、基础设施、生态环保、防灾减灾等领域补短板力度。

加快重点领域改革。持续深化“放管服”改革，推广“马上办”“最多跑一次”以及“证照分离”、事中事后综合监管等做法，以政府职能的转变带来市场活力的迸发。全面实行“互联网+政务服务”，加快推进政务信息系统整合共享，建立全市“一口录入、多部门共享”“一次登录、一网通办”的综合公共服务平台，实现与实体政务大厅有机衔接。规范公共资源交易平台建设，提高公共资源配置效率。开展国有资产清查工作，加强和改进国有资产监管。全面实施市场准入负面清单，清理废除妨碍公平竞争的各种规定和做法，支持民营经济发展。鼓励同等条件下优先使用本地企业产品。深化财税体制改革，做好环保税等税制改革工作。加快矿业权储备交易平台建设，推动矿业经济发展。

更高水平推进开放合作。封闭只会停滞倒退，开放才有活力进步。必须增强开放意识，通过开放促进发展、了解外界，也让外界了解乌兰察布。加快乌大张合作区建设，推进合作区规划修编工作。主动融入京津冀协同发展，密切与北京的对口帮扶合作关系，推进京蒙合作北京园建设，抓紧制定承接北京8个区的产业转移规划。发挥“一带一路”建设的开放引领作用，建好七苏木中欧班列物流基地、万益物流园区和北方陆港园区保税库。改善贸易环境，吸引更多商户入驻新雅宝路商城。扩大对外贸易规模，进出口总额达到10亿美元。

各位代表！今年是改革开放40周年，我们将以更大决心多推有利于增添经济发展动力的改革，多推有利于促进社会公平正义的改革，多推有利于增强人民群众获得感的改革，努力扩大改革的受益面，切实做到老百姓关心什么、期盼什么，改革就要抓住什么、推进什么。

（六）打好污染防治攻坚战，构建绿色生态安全屏障

推进生态建设保护。完成国家重点生态建设工程21万亩。组织好道路、村庄、厂矿、园区、学校、机关、企业绿化工程，合理布局公园绿地、绿道绿廊和环城绿化带建设，增加绿化总量，提升绿化质量。扩大退耕还林还草和轮作休耕，加大禁牧休牧轮牧力度，依法打击毁坏城市绿地和森林草原违法行为，保护好来之不易的生态建设成果，为子孙后代留下可持续发展“绿色银行”。

坚决有力治理污染。持续抓好中央、自治区环保督察整改工作，全面落实大气、水、土壤污染防治计划。做好全国第二次污染源普查工作。全面开展燃煤散烧集中治理，继续加大10蒸吨以下燃煤小锅炉淘汰力度，实施集宁区燃煤改气改电供暖工程。4家火电企业完成超低排放改造。全面推进河长制、湖长制，实现重点河湖依法管理保护全覆盖。开展岱海湖水质提升工程和缓冲带修复、重建等系统整治工程。加强黑河污染治理和出境断面水质达标考核。提升城市和工业污水处理及中水回用水平，加大城市饮用水水源地保护力度，城市污水处理厂全部达到一级A排放标准，中心城镇和园区污水资源化利用率达到50%。全面开展土壤污染调查，分类制定实施治理措施。

形成绿色发展方式。坚持空间监控一张蓝图，划定生态保护红线，落实国土主体功能区规划，明确各类保护区边界，推进严格监管常态化。严格控制开发强度，实施碳排放、能源消耗、水资源消耗、建设用地等总量和强度“双控”，多为生态“留白”，多给自然“种绿”。全面实施自然资源资产离任审计制度，领导干部离任先算“生态账”。强化环保执法，推动部门联防联控。实施全民节能行动计划。加强生态文明教育，让绿色消费、绿色生活成为时尚，让尊重自然、保护自然成为习惯。

（七）践行以人民为中心的发展思想，持续增进民生福祉

努力实现充分就业。坚持就业优先战略和积极就业政策，加强就业公共服务，统筹推进高校毕业生、零就业家庭、农牧业转移人口、复转军人等重点群体就业创业。支持市内高校、职业院校建设双创基地、实训基地，实施“大学生乌兰察布创业就业圆梦行动”。大数据服务外包产业实现直接就业2万人。加快市众创大厦等创业平台建设，培育发展众创、众包、众扶、众筹新业态新模式，实现创业带动就业。大力发展劳务经济，做大做强“乌兰美”等劳务品牌。加大对拖欠工程款和农民工工资行为的治理力度，构建和谐劳动关系。农村牧区劳动力转移就业37.5万人。

完善社会保障体系。全面实施全民参保计划。引导和鼓励进城务工、被征地农牧民参加职工养老保险。进一步完善职工、居民医保和大病保险制度，扩大覆盖范围，落实城乡居民就近参保和新生儿免费参保政策。开通城乡医保手机自助缴费系统。开展以单病种付费为主的复合型付费方式改革，减轻群众就医负担。扩大异地就医定点医院范围。完善社会救助和社会福利体系。提高社会救助对象认定精准度和救助标准，按户施保比例达到全区平均水平。提高孤儿、困境儿童、特困供养人员、残疾人等困难群体和高龄老人、优抚对象补贴标准，筑牢兜底保障网。支持和规范社会慈善捐助。深化殡葬改革，加快殡葬基础设施建设。

优先发展教育事业。继续深化教育改革，着力解决群众关心的热点难点问题，努力办好人民满意教育。实施义务教育优质均衡发展，全面提高教育教学质量。多措并举发展学前教育，毛入园率达到96%。规范幼儿园办学行为，让每个孩子健康快乐成长。加快普通高中优质特色多样化发展。坚持产教融合、校企合作，完善职业教育和培训体系，打造大数据等发展急需的品牌专业。优先发展民族教育，重视特殊教育，支持规范民办教育，办好继续教育，加快高等教育内涵发展。加强师德师风建设，提高教师素质。

提升医疗服务水平。深化医药卫生体制改革，破除以药养医。借助京蒙对口帮扶，采取专科共建、专家巡诊、医联体、整体托管等形式，加强与北京知名医院合作，让老百姓在家门口就能享受优质医疗服务。各旗县市区综合医院全部创建成二甲医院。推进名医工作室进社区，形成“小病进社区、大病转医院、康复回社区”的就医格局。在无村医的边远嘎查村全部实行“小药箱”工程。坚持中西医并重，推动蒙中医药事业传承发展。深入开展爱国卫生运动，做好公共卫生和重大疾病防控。改革完善计划生育服务管理，促进生育政策与相关经济社会政策的衔接。

加快文化事业发展。认真践行社会主义核心价值观，加强公民思想道德建设。加快市县两级图书馆、文化馆、美术馆、群艺馆、科技馆等场馆建设。推动乌兰牧骑事业发展，加强艺术精品创作，广泛开展群众性文化活动。加大文物、文化遗产、非遗保护力度。设立报刊阅读网点和“城市书房”，推进全民阅读。

各位代表！习近平总书记在新年贺词中指出，幸福都是奋斗出来的。我们要立足新时代民生需求的新变化，时刻把老百姓的安危冷暖放在心上，时刻把人民对美好生活的向往作为奋斗目标，想群众之所想，急群众之所急，既量力而行又尽力而为，切实办好每一件民生实事，以实打实、心贴心的举措造福于民。

（八）打好防范化解重大风险攻坚战，建设平安乌兰察布

防控财政金融风险。加强政府债务管理，打好预算安排、债权置换、资产重组、资源盘活组合拳，稳妥有序化解存量债务。强化预算约束，坚持量力而行、量入为出，严格规范地方政府投资项目、举债融资、基金运作和购买公共服务行为，从严控制政府债务增量。人大未批准、预算未安排、没有资金来源的新上项目，一律不予审批。推动融资平台公司公开透明、合法合规运作。坚持终身问责、倒查责任，加强重点督查和审计，坚决查处违法违规举债行为。加强金融风险防控，降低农信社等金融机构不良贷款率，强化互联网金融监管，综合治理农村牧区高利贷，确保不发生系统性区域性风险。

筑牢安全生产防线。“生命高于一切，安全重于泰山”。必须始终绷紧安全生产这根弦，牢固树立红线意识，坚持党政同责、一岗双责、企业主责、失职追责，以负面清单倒逼责任主体强化安全意识。深入开展重点领域重点行业常态化大排查大整治，坚决遏制重特大安全事故发生。健全突发事件应急处置机制，加强应急指挥体系建设，提高预测预警和防灾减灾能力。

加强社会综合治理。构建立体化、信息化社会治安防控体系，严密防范、依法打击各类犯罪活动，增强人民群众安全感。发挥综治基层网络和网格员作用，健全社会矛盾纠纷排查和调处机制，加强和改进信访工作，妥善解决好信访突出问题。完善城乡社区治理，建立“三社联动”机制，推动社会治理重心下移，更好回应社区居民多样化、个性化服务需求。

促进社会和谐共建。深化国防动员和双拥共建，促进军民融合发展。全面贯彻党的民族宗教政策，深入开展民族团结进步教育和创建活动。注重发挥老干部、工商联、工会、共青团、妇联、红十字会、科协、文联等组织的桥梁纽带作用，认真做好统计、地震、气象、档案等工作，推动各项社会事业再上新台阶。

四、努力建设人民满意的服务型政府

新时代要有新气象，更要有新作为。新一届政府要以党的十九大精神为指引，永葆蓬勃朝气，永做人民公仆，以实实在在的成效赢得人民群众的满意和点赞。

一要忠诚坚定,从严治政。旗帜鲜明讲政治，牢固树立“四个意识”，严守政治纪律和政治规矩，自觉在思想上政治上行动上同以习近平同志为核心的党中央保持高度一致。坚持党的领导，强化党组执行力，压实党建主体责任，加强党组自身建设，改善党组工作方法，把全面从严治党各项规定落实到政府工作各领域和全过程。

二要厉行法治，依法行政。牢固树立法治思维，切实把政府工作纳入法治轨道，全面推行行政权力清单化管理，严格按程序决策、按规矩办事。自觉接受人大、政协监督，认真办理人大代表议案、建议和政协委员提案，主动接受社会和舆论监督。积极推行政府法律顾问、公职律师制度，实现重大事项合法性审查全覆盖。加大政务公开力度，让权力在阳光下运行。

三要狠抓落实，高效施政。强化诚信意识，新官必须理旧账，以政务诚信建设带动全社会诚实守信，让老百姓和投资者有安全感、有信心。强化担当意识，把抓落实作为工作的生命线，实字当头、干字为先，不驰于空想，不骛于虚声，一步一个脚印，踏踏实实干好工作。强化责任意识，加强和改进督查考核问责工作，进一步完善正向激励和容错纠错机制，让改革者轻装上阵、担当者脱颖而出、实干者大显身手。

四要干净自律，廉洁从政。始终把廉洁从政作为政府工作的底线，坚定不移把反腐倡廉推向深入。严格执行中央八项规定实施细则，扎实推进“两学一做”学习教育常态化制度化，深入开展“不忘初心、牢记使命”主题教育，驰而不息纠“四风”，锲而不舍改作风。牢固树立“过紧日子”思想，坚持勤俭办一切事业，从严安排一般性支出，切实压缩行政经费支出。坚决查纠各种不正之风，努力营造风清气正的政务环境。

各位代表，风正潮涌，自当扬帆破浪；任重道远，更需快马加鞭。让我们更加紧密地团结在以习近平同志为核心的党中央周围，在自治区党委、政府和市委的坚强领导下，团结依靠全市各族人民，不忘初心、牢记使命，抢抓机遇、奋勇争先，在新的历史起点上，拥抱新时代，开启新征程，续写新辉煌，为决胜全面建成小康社会、夺取新时代中国特色社会主义伟大胜利而努力奋斗！
